# Combination Treatment of Perioperative Rehabilitation and Psychoeducation Undergoing Thoracic Surgery

**DOI:** 10.1155/2017/4743952

**Published:** 2017-02-09

**Authors:** Kazuhiro Hayashi, Takayuki Inoue, Motoki Nagaya, Satoru Ito, Hiroki Nakajima, Keiko Hattori, Izumi Kadono, Kohei Yokoi, Yoshihiro Nishida

**Affiliations:** ^1^Department of Rehabilitation, Nagoya University Hospital, Nagoya, Japan; ^2^Department of Respiratory Medicine, Nagoya University Graduate School of Medicine, Nagoya, Japan; ^3^Department of Orthopaedic Surgery, Nagoya University Graduate School of Medicine, Nagoya, Japan; ^4^Department of Thoracic Surgery, Nagoya University Graduate School of Medicine, Nagoya, Japan

## Abstract

Postoperative pulmonary complications are a risk associated with thoracic surgery. However, there have been few reports on cases at high risk of postoperative complications. Cancer patients often have negative automatic thoughts about illness, and these negative automatic thoughts are associated with reduced health behavior and physical activity. This case series demonstrates the successful combination treatment of perioperative rehabilitation and psychoeducation for negative automatic thoughts in two cancer patients who underwent thoracic surgery. One patient underwent pneumonectomy with laryngeal recurrent nerve paralysis; the other patient, who had a history of recurrent hepatic encephalopathy and dialysis, underwent S6 segmentectomy. Both patients had negative automatic thoughts about cancer-related stress and postoperative pain. The physical therapists conducted a perioperative rehabilitation program in which the patients were educated to replace their maladaptive thoughts with more adaptive thoughts. After rehabilitation, the patients had improved adaptive thoughts, increased physical activity, and favorable recovery without pulmonary complications. This indicates that the combination treatment of perioperative rehabilitation and psychoeducation was useful in two thoracic cancer surgery patients. The psychoeducational approach should be expanded to perioperative rehabilitation of patients with cancer.

## 1. Introduction

Although thoracic surgery is necessary for the treatment of cancer, it carries the risk of postoperative pulmonary complications [1–3]. Perioperative rehabilitation strategies recommended to prevent postoperative complications include lung expansion interventions, deep breathing exercises, incentive spirometry, and ambulation [1, 2].

Negative automatic thoughts are associated with reduced health behavior and physical activity [4, 5]. The psychoeducation for negative automatic thoughts reportedly enhances the adaptive coping and psychological wellbeing of cancer patients through the provision of medical and psychological information and the development of patient problem solving skills [6, 7]; this approach has been investigated starting several days after surgery [6, 7] and after the initiation of immunotherapy [6]. Negative automatic thoughts in cancer-related stress are expressed as helplessness, anxious preoccupation, fatalism, and avoidance [8, 9]. Negative automatic thoughts related to pain have also been successfully treated using the psychoeducation in the perioperative period in orthopedic surgery [10, 11]. Pain-related negative automatic thoughts are expressed as helplessness, rumination, and magnification [12–14]. Although the psychoeducational approach has been proven effective for negative automatic thoughts [6, 7, 10, 11, 15], further investigation is needed into the benefit of psychoeducational approaches in cancer rehabilitation.

Herein, we report two cases in which patients with negative automatic thoughts underwent thoracic cancer surgery. These patients received the combination treatment of perioperative rehabilitation and psychoeducation for negative automatic thoughts.

## 2. Case Presentation

### 2.1. Treatment

Treatment was provided by attending thoracic surgeons, anesthesiologists, nurses, and physical therapists. During the preoperative period, the patients received information about the operation and rehabilitation. After the operation, the patients were immediately extubated and transferred to intensive care unit. The patients started deep breathing, sitting, and standing by postoperative day 1 (POD1). Gait training was initiated after the patients returned to the general ward.

### 2.2. Rehabilitation Program

The rehabilitation program, which included psychoeducation, was conducted from the time of admission (a few days before surgery) until discharge. The most important treatment goal was the prevention of postoperative pulmonary complications. Perioperative rehabilitation, including orientation and measurements, was performed by a specialized physical therapist (K.H.) with 5-year experience who was trained in psychoeducation and cognitive behavior therapy. Training consisted of guided discovery, identifying and countering negative automatic thoughts, pacing, graded activity, relaxation, and other skills. During the preoperative period, the physical therapist provided medical and psychological information specific to cancer and the perioperative condition, as well as the plan for postoperative rehabilitation. The goal of providing psychological information was to help the patients understand the influence of various stressors on physical and mental conditions and to motivate them to change their behavior to reduce stress [7].

Patients were guided to explore their individual emotions, automatic thoughts, and beliefs based on Beck's approach [15]. The therapist helped the patients to distinguish their emotions and thoughts from an external event. The patients were helped to identify particular cognitive distortions and to replace these negative automatic thoughts and dysfunctional attitudes with more adaptive thoughts. The patients were educated in coping style and were encouraged to participate in more activities. The physical therapists went to the patients' rooms several times a day for the first 3-4 days after the operation and checked their exercise level and thoughts. The presence of the physical therapists was then gradually reduced to facilitate independence. The 6-minute walking distance (6MWD) measured by the 6-minute walking test was performed during the preoperative period and at discharge by following a standardized procedure in room air [16]. Publication of these cases was approved by the ethics committee of Nagoya University Hospital (number 2015-0413).

## 3. Case 1

### 3.1. Pneumonectomy with Recurrent Nerve Paralysis

#### 3.1.1. Patient History

The patient was a 60-year-old man. He had a high school degree, lived alone, and worked until he was 60 years old. He smoked 30 cigarettes per day for 20 years until he was 40 years old. He had a history of moderate alcohol consumption for 20 years until he was 40 years old. He did not regularly exercise. This patient was referred to our university hospital for investigation of a cough with bloody sputum that had persisted for months. He had no other comorbidities.

Chest CT revealed a 6.3 × 5.2 cm mass located in the left central portion of the lung and enlarged hilar lymph nodes. The tumor was diagnosed as stage IIB (T2bN1M0) squamous cell carcinoma 6 months after the patient first experienced coughing symptoms.

Clinical findings on admission were normal, except that electrocardiography revealed sinus tachycardia (heartrate > 110 bpm) (Table 1). The 6MWD immediately after admission was 493 m. The patient's thoughts regarding his illness were as follows: “There is nothing I can do to help myself” and “I have put myself at the doctor.” These thoughts were similar to one of the items in the helplessness and fatalism maladaptive coping style [8, 9].

#### 3.1.2. Perioperative Course

Medical and psychological information was provided during the preoperative period, and the modifying structured association counseling technique was performed throughout the study [7]. The patient was provided with opportunities to remember how he had lived before his cancer diagnosis and was encouraged to be aware of how he was being affected by the stress of the cancer. The psychoeducation and information consisted of “stress and both physical and psychological reaction,” “stress and its related factors (including personality, self-esteem, and social support),” and “better attitude for living (including having a positive attitude, expressing negative emotions, getting social support, and the effectiveness of laughter).” The physical therapists helped the patient find ways to manage stressful situations and to better perform health behavior and physical activity.

The patient underwent a left pneumonectomy and combined resection of the left recurrent nerve. Operating time was 198 minutes, and blood loss was 105 mL. Postoperative diagnosis was stage IIIA squamous cell carcinoma. The patient had tachycardia (heartrate > 110 bpm) and was given landiolol hydrochloride (3.39 *μ*g/kg/min) on POD1. A tracheal tube was inserted via cricothyrotomy due to insufficient sputum expectoration. The patient returned to the general ward on POD2. Postoperative pain was managed with continuous epidural anesthesia, patient-controlled analgesia, and oral loxoprofen sodium. Oral intake without aspiration was possible on POD4. The patient's thoughts were “I suffer from a lot of anxiety due to my disease,” “I have difficulty in believing this happened to me,” and “I distract myself when thoughts about my illness come into my head”; these thoughts were similar to one of the items in the anxious preoccupation and avoidance maladaptive coping style [8, 9]. However, the patient also thought “I try to fight the illness,” which was similar to one of the items in the fighting spirit of the adaptive coping style [8, 9].

The patient could independently perform activities of daily living and was discharged home on POD16. The 6MWD at discharge was 455 m. The patient thought “I am determined to beat this disease” and “I see my illness as a challenge,” which was similar to one of the items in the fighting spirit of the adaptive coping style [8, 9]. After discharge, the patient regularly walked 30 minutes daily to improve physical function. The patient was not readmitted for any pulmonary complications.

## 4. Case  2

### 4.1. S6 Segmentectomy with Comorbidities of Hepatic Encephalopathy and Dialysis

#### 4.1.1. Patient History

The patient was a 67-year-old man with hypertension who had undergone pancreatoduodenectomy for pancreatic cancer 7 years ago. He also had noncirrhotic recurrent hepatic encephalopathy after shunting from the superior mesenteric vein to the inferior vena cava for a jejunal varicose vein for 3 years and dialysis for chronic kidney disease for 1 year. He had a high school degree, lived with family, and worked until he was 60 years old. He smoked 40 cigarettes per day for 30 years until he was 50 years old. He had no history of alcohol consumption. He did not regularly exercise.

Chest CT screening after pancreatoduodenectomy in our hospital revealed a 1.1 × 1.4 cm mass located in the right S6. The tumor was diagnosed as stage IV (T1aN0M1) lung metastasis of pancreas adenocarcinoma. The patient was advised to limit the use of postoperative analgesic drugs because of comorbidities. He had no signs of pleural effusion, ascites, disturbance of consciousness, or obvious fatigue. Clinical findings on admission were normal, except that the pulmonary function test revealed a mild restrictive defect (Table 1). The 6MWD on admission was 372 m.

Preoperative pain-related psychological findings were measured using the Pain Catastrophizing Scale (PCS), which consists of 13 items and gives a score ranging from 0 to 52 [12, 14]. The PCS measures a subject's perception of how frequently they have experienced negative automatic thoughts and predicts postoperative pain [17, 18]. The total PCS score was 23, and the subscales were 15 in rumination, 5 in magnification, and 3 in helplessness. The total PCS score was above the cutoff points of strong pain prediction, which have been reported to be 13 [18] or 16 [19]. The patient's thoughts were “I cannot seem to keep pain out of my mind” and “I keep thinking about how badly I want the pain to stop,” which was one of the items in the rumination of pain of the maladaptive coping style [12–14].

#### 4.1.2. Perioperative Course

Medical and psychological information was provided during the preoperative period, and training to modify pain coping skills was performed throughout the study [11]. The patient was provided with opportunities to recognize pain-related negative automatic thoughts, such as pain catastrophizing, and to replace these with alternative, rational, reassuring, adaptive thoughts.

#### 4.1.3. Postoperative Course

The patient underwent S6 segmentectomy of the right lung. The operating time was 87 minutes, and blood loss was 103 mL. The patient returned to the general ward on POD1. Postoperative analgesic drugs included continuous fentanyl (50 *μ*g/hr) until POD2 and oral celecoxib (up to 200 mg per day as needed). Continuous epidural anesthesia and patient-controlled analgesia were not used because of comorbidities. Pain intensity using a 0–10 numerical rating scale was 8/10 on POD1, 5/10 on POD2, 3/10 on POD3 and POD4, and 0–2/10 on all subsequent days. The patient thought “I do something active, which diverts my attention away from the pain” and “I tell myself to be brave and carry on despite the pain,” which was similar to one of the items in the adaptive coping style [13]. Oral intake without aspiration was possible on POD6.

The patient was discharged home on POD19. The 6MWD at discharge was 344 m. The patient thought “I see my illness as a challenge,” which was similar to one of the items in the fighting spirit of the adaptive coping style [8, 9]. After discharge, the patient regularly walked for 20 minutes every day to improve his physical function. The patient was not readmitted for any pulmonary complications.

## 5. Discussion

The present patients had negative automatic thoughts and were assessed as having high risk of postoperative pulmonary complications following thoracic surgery. These patients received combination treatment of perioperative rehabilitation and psychoeducation, which led to improved adaptive thoughts, increased physical activity, and favorable recovery.

Psychological intervention should be based on the individual patient's specific goals for problem solving, not just a general coping strategy [7]. Negative automatic thoughts are associated with reduced health behavior and physical activity [4, 5]. Therefore, perioperative rehabilitation for cancer patients should focus not only on exercise, but also on psychoeducation for coping style. Successful exercise experiences can dramatically improve negative automatic thoughts. Physical therapists often initiate postoperative exercise, and so they should take the lead in psychoeducation to help patients replace negative thoughts with more adaptive coping styles during rehabilitation. As psychological constructs and QOL are worse in patients with short duration rather than long duration since diagnosis [20–22] and surgery [23–25], early combination treatment of rehabilitation and psychoeducation could be useful in cancer patients. Patients in the early postoperative phase experience pain and reduction of QOL, and therefore the treatment should be individualized for each patient's symptoms.

Cancer patients often have psychological concerns and maladaptive coping styles about illness in the period from diagnosis to the completion of treatment [26, 27], as observed in Case  1. Perioperative psychological and physical changes occur before the patient is discharged. Hence, psychoeducation for effective coping strategies should be performed constantly during the perioperative period. Previous studies have reported that surgical patient outcomes are improved by patient education [28, 29], and the maladaptive coping style is a risk factor for psychiatric morbidity and decreased survival in cancer patients [26, 30–32]. Further longitudinal psychological assessment and treatment are required from diagnosis to completion of treatment in cancer patients.

The patient in Case  2 had a high PCS score and marked postoperative pain. Preoperative pain catastrophizing predicts persistent postoperative pain in total joint replacement patients [19, 33, 34]. Marked postoperative pain prevents effective cough-up and deep breathing and increases the risks of postoperative complications [35]. Positive coping for pain, such as thinking of things to distract them from the pain or engaging in active behaviors that divert attention away from the pain, is useful for health behavior [13]. The psychoeducation for the maladaptive coping style in pain should be performed perioperatively.

This case report has several limitations. The negative automatic thoughts were assessed mainly by medical interview. Several short screening tools about negative automatic thoughts have been reported [36]. Development of simple psychological assessment tools for perioperative cancer patients is required.

## 6. Conclusions

The combination treatment of perioperative rehabilitation and psychoeducation is useful for improving automatic thoughts and behaviors about illness and leads to favorable recovery in thoracic cancer surgery. The psychoeducational approach should be expanded to perioperative cancer patients.

## Figures and Tables

**Table 1:** Patient characteristics and response to treatment.

	Case 1	Case 2
Age	60-year-old	67-year-old
Sex	Male	Male
Comorbidity	No	Hepatic encephalopathy and dialysis
Laboratory values on admission		
Albumin (g/dl)	3.7	2.6
Hemoglobin (g/dl)	12.7	9.8
Respiratory function test on admission		
VC (L)	3.62	2.99
% VC (%)	100.6	79.4
FEV_1_ (L)	2.74	2.90
FVC (L)	3.79	2.97
FEV_1_/FVC (%)	72.2	89.2
Electrocardiography on admission	Sinus tachycardia	Normal
Surgery	Pneumonectomy and combined resection with recurrent nerve	S6 segmentectomy
6MWD (m)		
on admission	493	372
at discharged	455	344
Statement		
on admission	There is nothing I can do to help myself. (helplessness) I have put myself in the doctor. (fatalism)	I cannot seem to keep pain out of my mind. (rumination) I keep thinking about how badly I want the pain to stop. (rumination)
immediately after surgery	I try to fight the illness. (fighting spirit) I suffer a lot of anxiety due to my disease. (anxious preoccupation) I have difficulty in believing this happened to me. (anxious preoccupation) I distract myself when thoughts about my illness come into my head. (avoidance)	I do something active which divert one's attention away from the pain. (increasing activity level) I tell myself to be brave and carry on despite the pain. (coping selfstatement)
at discharged	I am determined to beat this disease. (fighting spirit) I see my illness as a challenge. (fighting spirit)	I see my illness as a challenge. (fighting spirit)

VC, vital capacity; % VC, percent vital capacity; FEV_1_, forced expiratory volume in 1 second; FVC, forced vital capacity; 6MWD, 6-minute walking distance.
